# Evidence of Interspecific Chromosomal Diversification in Rainbowfishes (Melanotaeniidae, Teleostei)

**DOI:** 10.3390/genes11070818

**Published:** 2020-07-18

**Authors:** Zuzana Majtánová, Peter J. Unmack, Tulyawat Prasongmaneerut, Foyez Shams, Kornsorn Srikulnath, Petr Ráb, Tariq Ezaz

**Affiliations:** 1 Laboratory of Fish Genetics, Institute of Animal Physiology and Genetics, Czech Academy of Sciences, 27721 Liběchov, Czech Republic; rab@iapg.cas.cz; 2 Centre for Applied Water Science, Institute for Applied Ecology, University of Canberra, Bruce, ACT 2617, Australia; peter.unmack@canberra.edu.au; 3 Laboratory of Animal Cytogenetics and Comparative Genomics (ACCG), Department of Genetics, Faculty of Science, Kasetsart University, Bangkok 10900, Thailand; tulyawat_whale@hotmail.com (T.P.); kornsorn.s@ku.ac.th (K.S.); 4 Centre for Conservation Ecology and Genetics, Institute for Applied Ecology, University of Canberra, Bruce, ACT 2617, Australia; foyez.shams@canberra.edu.au (F.S.); tariq.ezaz@canberra.edu.au (T.E.)

**Keywords:** karyotype, rDNA, chromosome, variability, FISH, *Melanotaenia*, *Iriatherina*, *Glossolepis*, *Rhadinocentrus*, *Cairnsichthys*

## Abstract

Rainbowfishes (Melanotaeniidae) are the largest monophyletic group of freshwater fishes occurring in Australia and New Guinea, with 112 species currently recognised. Despite their high taxonomic diversity, rainbowfishes remain poorly studied from a cytogenetic perspective. Using conventional (Giemsa staining, C banding, chromomycin A_3_ staining) and molecular (fluorescence in situ hybridisation with ribosomal DNA (rDNA) and telomeric probes) cytogenetic protocols, karyotypes and associated chromosomal characteristics of five species were examined. We covered all major lineages of this group, namely, Running River rainbowfish *Melanotaenia* sp., red rainbowfish *Glossolepis* *incisus*, threadfin rainbowfish *Iriatherina werneri*, ornate rainbowfish *Rhadinocentrus ornatus*, and Cairns rainbowfish *Cairnsichthys rhombosomoides*. All species had conserved diploid chromosome numbers 2n = 48, but karyotypes differed among species; while *Melanotaenia* sp., *G. incisus*, and *I. werneri* possessed karyotypes composed of exclusively subtelo/acrocentric chromosomes, the karyotype of *R. ornatus* displayed six pairs of submetacentric and 18 pairs of subtelo/acrocentric chromosomes, while *C. rhombosomoides* possessed a karyotype composed of four pairs of submetacentric and 20 pairs of subtelo/acrocentric chromosomes. No heteromorphic sex chromosomes were detected using conventional cytogenetic techniques. Our data indicate a conserved 2n in Melanotaeniidae, but morphologically variable karyotypes, rDNA sites, and heterochromatin distributions. Differences were observed especially in taxonomically divergent species, suggesting interspecies chromosome rearrangements.

## 1. Introduction

The genomes of teleost fishes display remarkable features (e.g., variability in size or basic chromosome organisation) that might be involved in the formation of their immense species diversity. Access to whole-genome sequences provides important insights into the gene occurrence and organisation within a species, and it revolutionises our understanding of how genetic information is stored and organised in DNA molecules and chromosomes and how it evolved over time. However, even high-quality whole-genome sequencing does not offer a complete picture of the genome. This is mainly caused by difficulties with assembling repetitive DNA, sequences important for the structural and functional organisation of the genome [1]. In this respect, teleost fish cytogenetics and cytogenomics represent powerful tools in determining genome organisation. Repetitive DNA were localised on chromosomes of hundreds of teleost fish species so far, helping to understand chromosomal variation and genome organisation [2,3,4]. Although chromosomes of teleost fish are overall widely studied, the cytogenetic and cytogenomic research of Australian fishes is almost absent. Taking into account the high Australian freshwater fish diversity, the study of chromosomes is important for understanding the role of chromosome evolution in their speciation.

The freshwater fish fauna in Australia and New Guinea consists of approximately 40 families with a total number of species exceeding 500 [5]. Among them, the family Melanotaeniidae, commonly known as rainbowfishes, represents the most speciose family of freshwater fishes in this region with the total number of 112 species [6] and at least an additional 15–20 undescribed species [7]. Along with gobiids (Gobiidae) and eleotrids (Eleotridae), rainbowfishes are one of the most abundant families in Australia and New Guinea [7]. Phylogenetic relationships among rainbowfish species were analysed in several molecular systematic studies so far [7,8,9,10] with nearly concurrent conclusions. Rainbowfishes consist of seven genera, of which three are monotypic (*Iriatherina*, *Pelangia* and *Rhadinocentrus*) and one has two species (*Cairnsichthys*). The remaining three lineages are more speciose including *Melanotaenia* (87 spp.), *Chilatherina* (11 spp.), and *Glossolepis* (9 spp.) [6,7].

Rainbowfishes are relatively small fishes (mostly <10 cm) which are extremely popular in the aquarium trade due to their bright male colouration and ease of breeding and captive care [11]. They are ecological generalists, which allows them to be one of the most widespread freshwater fish groups in Australia and New Guinea. They occur in virtually all freshwater habitats from large tropical rivers, lowland and medium gradient rainforest streams, lakes, and floodplain wetlands to isolated desert waterholes and more temperate environments. While mostly found in lower elevations up to 1000 m or so (most of Australia lacks elevation >1000 m), a few species occur up to 1800 m [12]. It is not uncommon that up to four rainbowfish species occur sympatrically, while the more arid or temperate parts of their range host mostly only a single species. Active hybridisation between sympatric species is quite rare, although there is plenty of evidence for older introgression between some species [7]. Introgression is only detected between more closely related rainbowfish lineages. Three of the more distantly related genera (*Cairnsichthys*, *Rhadinocentrus*, and *Iriatherina*) were not found to hybridise in wild populations, despite often sharing habitats with another rainbowfish species. This suggests that divergent chromosomal evolution may be playing a role of the postzygotic barrier in hybridisation events (as shown in, e.g., Molina et al. [13]).

Despite the general interest in this group, rainbowfishes are poorly studied cytogenetically. The diploid chromosome number (2n) and karyotypes are known for only nine out of the 112 extant species [14,15,16,17,18]. In these studied species, the diploid chromosome number is reported to be conserved (2n = 48) with the only exceptions being *Melanotaenia maccullochi* (2n = 46 or 48) and *Melanotaenia lacustris* (2n = 46) [15,16,17,18]. The karyotypes were mostly studied using conventional cytogenetic techniques, with no images published. In this study, we focused on representatives of five main phylogenetic lineages among rainbowfishes to cover trends in chromosome and genome organisation in this group. Using molecular cytogenetic methods, we examined Running River rainbowfish (*Melanotaenia* sp.), red rainbowfish (*Glossolepis incisus*), threadfin rainbowfish (*Iriatherina werneri*), ornate rainbowfish (*Rhadinocentrus ornatus*), and Cairns rainbowfish (*Cairnsichthys rhombosomoides*). Moreover, we reviewed all available cytogenetic data for the representatives of family Melanotaeniidae and combined previous findings with our results to interpret chromosome evolution and speciation.

## 2. Materials and Methods

### 2.1. Studied Material

We examined 25 individuals from five species as follows: three males and three females of *Melanotaenia* sp. (F_1_ captive bred fish from Running River, Queensland, Australia); five males and two females of *I. werneri* (obtained via the European aquarium trade); two males and two females from *G. incisus* (obtained via the Australian aquarium trade)*;* two males and two females from *R. ornatus* (large captive population originally from Spring Creek, Carindale, Queensland, Australia); two males and two females from *C. rhombosomoides* (wild fish from Ninds Creek, Queensland, Australia). Wild samples were obtained under state fishery permits, and research was conducted with approval from the University of Canberra Ethics Committee (CEAE.15-05). More information about the number of cells is listed in Table 1.

### 2.2. Chromosome Preparation and Staining

Metaphase chromosomes were prepared according to Bertollo et al. [19] with slight modifications. Briefly, fish were injected with 0.1% colchicine solution (1 mL/100 g of body weight) 45 min before being sacrificed using an overdose of anaesthetic (clove oil). The kidneys were dissected in 0.075 M KCl at room temperature. The cell suspension free of tissue fragments was hypotonised for 30 min in 0.075 M KCl, fixed in freshly prepared fixative (methanol: acetic acid 3:1, *v*/*v*), washed twice in fixative, and finally spread onto slides. Chromosomal preparations from all individuals were stained with conventional Giemsa solution (5%, 10 min) or DAPI (4′,6-diamidino-2-phenylindole) in order to confirm number and morphology of their chromosomes. C banding, visualising blocks of constitutive heterochromatin, was performed according to Sumner [20] with slight modifications described in Pokorná et al. [21]. After C-banding, the chromosomes were counterstained by DAPI to enhance the contrast, and the images were captured in the fluorescent regime and inverted. We applied reversed fluorescent staining with chromomycin A_3_ (CMA_3_) specific for GC-rich regions, counterstained with DAPI, with a higher affinity for AT-rich regions [22].

### 2.3. Fluorescence In Situ Hybridisation (FISH) with Telomeric and rRNA Genes Probes

The organisation of the telomeric motif (TTAGGG)_n_, as well as rDNA genes, within the genomes was analysed by fluorescence in situ hybridisation (FISH). Telomeric FISH was performed using a Cy3-labelled PNA (peptide nucleic acid) probe according to the manufacturer’s instructions (Telomere PNA FISH Kit/Cy3, Dako). Probes for FISH experiments were produced by PCR with primer pairs and thermal cycling conditions according to Komiya et al. [23] for 5S rDNA and Zhang et al. [24] for 28S rDNA. The PCR reactions were carried out in a final volume of 25 μL consisting of 100 ng of genomic DNA of *C. rhombosomoides*, 12.5 μL of PPP master mix, 0.01 mM of each primer, and PCR water to complete the volume (all reagents from TopBio, Prague, Czech Republic). Probes were indirectly labelled with biotin-16-dUTP (Roche, Mannheim, Germany) and digoxigenin-11-dUTP (Roche) through PCR reamplification of PCR products. Reamplification was carried out under the same conditions as the previous PCR reaction. Labelled PCR products were precipitated. The hybridisation mixture consisted of hybridisation buffer [25] and differently labelled PCR products of both genes. The hybridisation and detection procedures were carried out under conditions described in Symonová et al. [25]. The biotin-dUTP-labelled probes were detected by the Invitrogen Cy™3-Streptavidin (Invitrogen, San Diego, CA, USA; cat. no. 43–4315), while the digoxigenin-dUTP-labelled probes were detected by the Roche Anti-Digoxigenin-Fluorescein (cat. no. 11207741910). Finally, the slides were mounted with Vectashield DAPI anti-fade medium (Vector Laboratories, Burlingame, CA, USA).

### 2.4. Microscopy and Image Analyses

Chromosomal preparations were examined using an Olympus Provis AX 70 epifluorescence microscope (Olympus, Tokyo, Japan). Images of metaphase chromosomes were recorded with a cooled Olympus DP30BW CCD camera. The IKAROS and ISIS imaging programs (Metasystems, Altlussheim, Germany) were used to analyse grey-scale images. The captured digital images from FISH experiments were pseudocoloured (red for Anti-Digoxigenin-Rhodamine, green for Invitrogen FITC-Streptavidin) and superimposed using Adobe Photoshop software, version CS5. In the case of CMA_3_/DAPI staining, the CMA_3_ signal was inverted into the red and the DAPI signal into the green channel to enhance the contrast between these two types of signals. Chromosomes were ordered within each group (sm = submetacentric, st = subtelocentric, a = acrocentric) in decreasing size order.

## 3. Results

### 3.1. Karyotypes

**Figure 1:**
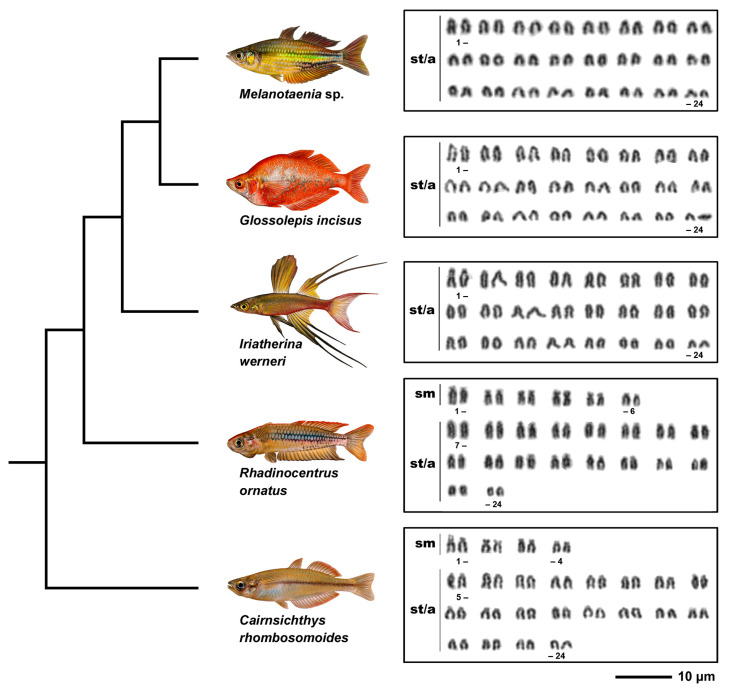
Phylogenetic relationships and karyotypes of studied species. Truncated phylogeny representing five species under study and their Giemsa-stained karyotypes. Phylogenetic relationships follow Unmack et al. [7]. sm, submetacentric; st/a, subtelocentric-acrocentric chromosomes. Bar = 10 µm.

Karyotypes of all five species examined possessed the diploid chromosome number 2n = 48 (Figure 1, Table 2). However, species differed in chromosome morphology and fundamental number (NF). *Melanotaenia* sp., *G. incisus*, and *I. werneri* possessed the same karyotype composed of exclusively 48 subtelo/acrocentric chromosomes and NF = 48, while the karyotype of *R. ornatus* was composed of six pairs of submetacentric and 18 pairs of subtelo/acrocentric chromosomes with NF = 60. *C. rhombosomoides* karyotype was composed of four submetacentric and 20 subtelo/acrocentric pairs of chromosomes with NF = 56. No intraspecific numerical or structural polymorphisms between males and females were observed in any of the study species (Figure S1, Supplementary Materials). We noticed differences in size of the first chromosome pair between *C. rhombosomoides* male and female, possibly associated with different accumulation of 28S rDNA copies in the p-arms of these chromosomes (Figure S1, Supplementary Materials).

### 3.2. rDNA Chromosome Mapping

**Figure 2:**
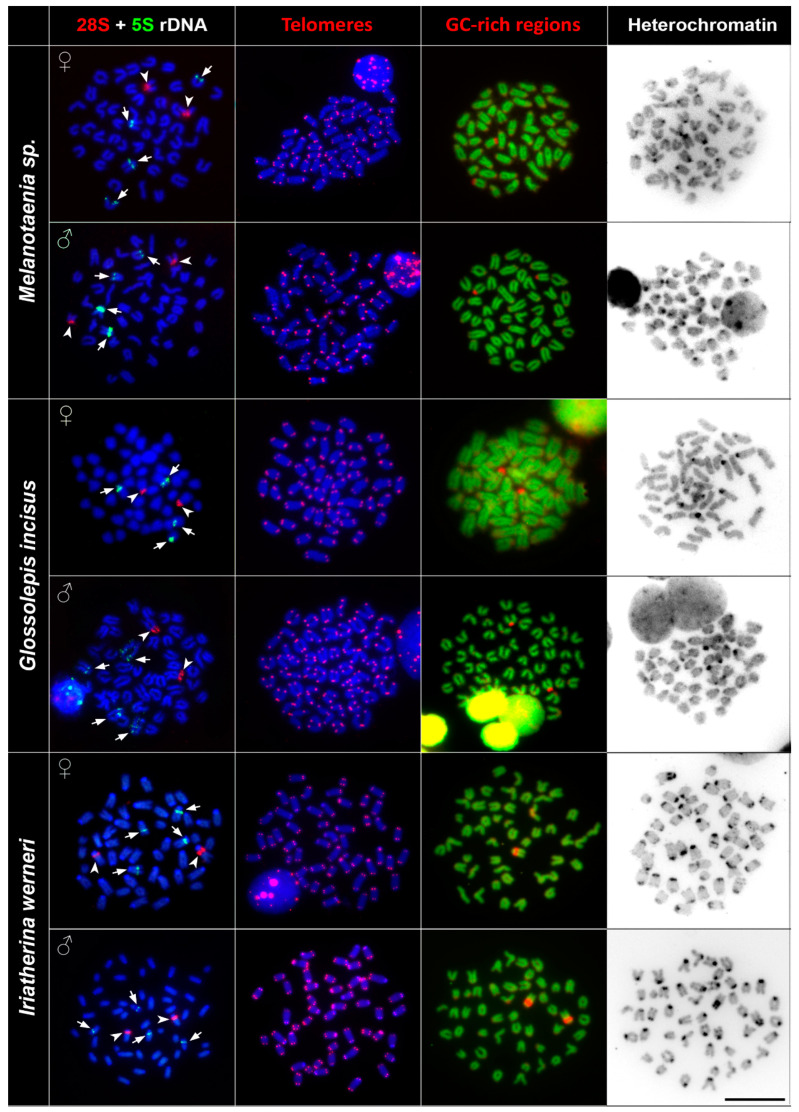
Comparative chromosome analyses of *Melanotaenia* sp., *G. incisus*, and *I. werneri*. First column: DAPI (4′,6-diamidino-2-phenylindole)-stained chromosomes (blue), 28S rDNA (red, indicated by arrowheads), 5S rDNA (green, indicated by arrows) hybridisation signals; second column: DAPI-stained metaphases (blue), telomere repeat hybridisation signals (red); third column: DAPI-stained metaphases (green), signals of GC-rich regions (red); fourth column: inverted DAPI-stained C banding pattern. Bar = 10 µm.

**Figure 3:**
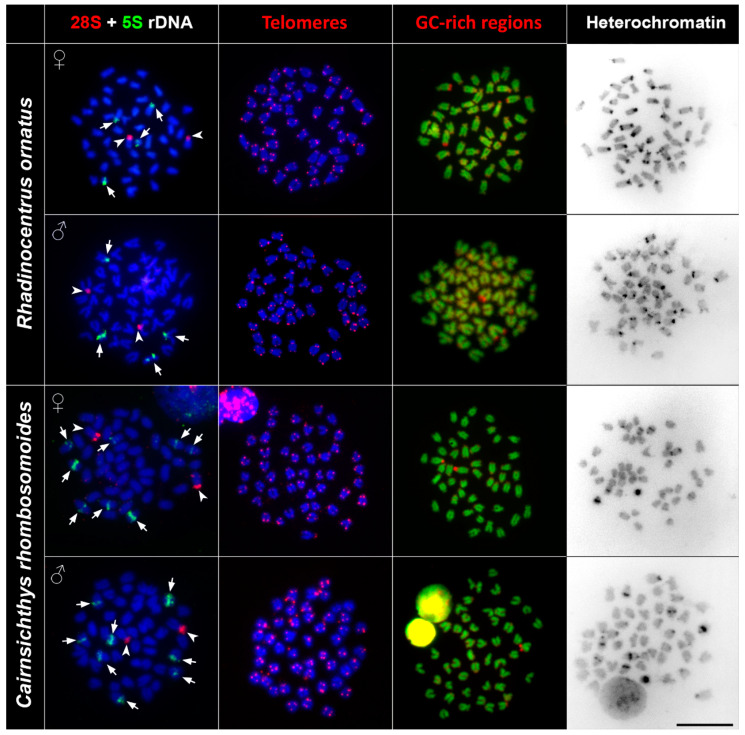
Comparative chromosome analyses of *R. ornatus* and *C. rhombosomoides*. First column: DAPI-stained metaphases (blue), 28S rDNA (red, indicated by arrowheads), 5S rDNA (green, indicated by arrows) hybridisation signals; second column: DAPI-stained metaphases (blue), telomere repeat hybridisation signals (red); third column: DAPI-stained metaphases (green), signals of GC-rich regions (red); fourth column: inverted DAPI-stained C banding pattern. Bar = 10 µm.

The FISH (fluorescence in situ hybridisation) experiments with 5S and 28S rDNA probes showed specific signals on non-homologous chromosomal pairs among the studied species. The 28S rDNA probe hybridised at the pericentromeric regions of one subtelo/acrocentric chromosomal pair in all species. The number of the 5S rDNA loci differed among studied species. *Melanotaenia* sp., *G. incisus*, *I. werneri*, and *R. ornatus* possessed a total four hybridisation signals of 5S rDNA, two on the telomeric region of one subtelo/acrocentric chromosomal pair, and two signals in the centromeric regions of the another subtelo/acrocentric chromosomal pair. *Cairnsichthys rhombosomoides* possessed the highest number of 5S rDNA copies (eight signals in total), with two signals located in the telomeric region of one subtelo/acrocentric chromosomal pair and six interstitial (between the telomeres and centromeres) signals on three other subtelo/acrocentric chromosomal pairs (Figure 2 and Figure 3).

### 3.3. Telomere Mapping

In order to document interstitial telomeric sequences (ITSs) as remnants of chromosomal rearrangements, we performed FISH with the conserved vertebrate telomeric repeat (TTAGGG)_n_. We detected signals at the termini of all chromosomes but did not detect any ITSs in any species examined (Figure 2 and Figure 3).

### 3.4. CMA_3_/DAPI Staining

Reversed fluorescence staining (CMA_3_/DAPI) revealed homogeneous staining patterns across chromosomes with a moderately GC-rich centromeric and telomeric regions. Extremely GC-rich signals were found in the centromeric region of one subtelo/acrocentric chromosomal pair in all but *R. ornatus.* In *R. ornatus,* strong GC-rich signals were located in the telomeric region of one subtelo/acrocentric chromosomal pair, corresponding to the signals revealed also by C banding (Figure 3). In addition to signals in telomeric regions, several CG-rich signals were observed in pericentromeric regions in both male and female *R. ornatus* (Figure 3).

### 3.5. C Banding

C banding revealed interspecies differences of constitutive heterochromatin among species. In *Melanotaenia* sp., we observed approximately 32 C-positive bands in centromeric regions, while, in *G. incisus*, there were 14 C-positive bands in centromeric regions. *I. werneri* displayed 36 C-positive bands in pericentromeric regions and four interstitial C-positive bands (Figure 2). In *R. ornatus* 28 C-positive bands in centromeric regions, in addition to two C-positive bands in telomeric regions of subtelo/acrocentric chromosomes, were observed. In *C. rhombosomoides* C banding showed eight C-positive bands in centromeric regions together with four interstitial C-positive bands and two subtelo/acrocentric chromosomes with large heterochromatin blocks (Figure 3). No sex-specific C banding pattern was observed in any species.

## 4. Discussion

Rainbowfishes represent one of the most widespread and abundant freshwater families in Australia and New Guinea. They became very popular among aquarium hobbyists such that they are now available in pet stores around the world [11]. Similarly, they attract the attention of the scientific world with wide-ranging studies focused on their ecology, biology, phylogeny, and conservation (e.g., McGuigan et al. [26], Page et al. [27], Colléter and Brown [28]). Despite the fact that cytogenetics provides a valuable source for understanding genome evolution using information undetectable by molecular genetics (e.g., Dion-Côté et al. [29]), chromosome studies of rainbowfishes are scarce. In five studies published so far, the chromosome number of nine species was reported, namely, *M. duboulayi* (2n = 48a) [17], *M. maccullochi* (2n = 46 or 2n = 48) [15,18], *M. fluviatilis* (2n = 48) [14], *M. goldiei* (2n = 48a) [14], *M.* cf. *splendida* (2n = 48) [16], *M. bosemani* (2n = 48) [18], *M. lacustris* (2n = 46) [18], *M. praecox* (2n = 48) [18], and *G. incisus* (2n = 48) [18]. Unfortunately, none of these studies included any images of karyotypes or detailed information on other chromosomal characteristics. Thus, it was not possible to compare these data with our results in more detail. Here, we described the chromosome number of five species representing the five major lineages of this family [7]. The Running River rainbowfish (*Melanotaenia* sp.) is representative of the “Australis” phylogenetic lineage which was until recently included within the *M. splendida* (eastern rainbowfish) complex [30]. Nevertheless, recent genetic analyses (P. Unmack unpub. data) using single-nucleotide polymorphisms (SNPs) determined that the Running River rainbowfish is an undescribed species. *Glossolepis incisus*, representing the northern lineage, is the only species in this study with known chromosome number as reported by Said [18]. However, this research was not published in English and provides only a statement about the chromosome number with no images of karyotypes depicting detailed chromosomal characteristics. *I. werneri*, *R. ornatus*, and *C. rhombosomoides* represent early branching lineages within the phylogeny of the family [7].

Previous cytogenetic analyses revealed uniform chromosome numbers across rainbowfish species (2n = 48) as the most parsimonious ancestral state for major teleostean clades [31,32]. The diploidy in rainbowfishes was probably maintained over long evolutionary time. In addition to the stable 2n, similarities in other chromosomal characteristics are presented in our study. The most closely related species, *Melanotaenia* sp. and *G. incisus*, with an estimated divergence from each other between 23.6 and 37.3 Mya [7] possessed similar karyotype characteristics, as did *I. werneri*, one of the early branching lineages with estimated divergence that pre-dates ~40 Mya [7]. These three species possessed the same karyotypes (Figure 1 and Figure S1, Supplementary Materials) and the same numbers of clusters of 5S and 28 rDNA genes (Figure 2). Moreover, other chromosomal markers displayed similar patterns, i.e., constitutive heterochromatin regions were located mostly in the centromeric regions and interstitial positions of chromosomes, mainly those associated with NOR (nucleolar organizer region) signals (Figure 2, Table 2). These phylogenetically shared chromosomal features can indicate similar levels and patterns of chromosomal evolution within a clade [33]. The hybridisation of species in the genera *Melanotaenia* and *Glossolepis* in captivity, and a lack of significant barriers to introgressive hybridisation [34] suggest that hybridisation may also be linked with karyotype stasis among these clades. There are examples of active hybridisation of representatives from these clades in nature where different widespread species are coming into reproductive contact at their boundaries with a mix of parental species, F1 hybrids, and backcrosses present [35]. Moreover, there are a number of examples where mitochondrial DNA (mtDNA) introgression was demonstrated without evidence of nuclear introgression. This usually occurs between sympatric species from different rainbowfish lineages [7]. The two other species under this study, *R. ornatus* and *C. rhombosomoides*, displayed species-specific differences at both karyotype and chromosomal marker levels, which could represent evidence of the chromosomal background of speciation. Specifically, they differ in the chromosomal morphology or in the number of rDNA sites; both species possess two pairs of 28S rDNA but four (*R. ornatus*) and eight signals of 5S rDNA (*C. rhombosomoides*) (Figure 3, Table 2). It is commonly accepted that major 28S rDNA clusters of teleostean fishes are GC-rich and, thus, can also be identified by CMA_3_ staining (e.g., Mayr et al. [36], Amemiya and Gold [37], Schmid and Guttenbach [38]). The same situation was observed in all species under this study with the exception of *R. ornatus.* In this species, the 28S rDNA clusters were only moderately GC-rich in addition to GC-rich signals located on non-homologous chromosomes. The lack of correspondence between CMA_3_+ regions and 28S rDNA loci may be explained by the lower copy number of rRNA genes, as already reported in fishes (e.g., Gromicho et al. [39], Sola et al. [40]). We observed no intraspecific numerical or structural polymorphisms between males and females, suggesting a lack of heteromorphic sex chromosomes (Figure 2, Figure 3 and Figure S1, Supplementary Materials). The only polymorphism was observed in the size of p-arms in the first chromosome pair in *C. rhombosomoides*, where unequal accumulation of 28S rDNA copies possibly led to slightly different size of chromosomes (Figure S1, Supplementary Materials). In all studied species, no ITSs were observed. ITSs sites are usually considered as relicts of the ancient chromosomal rearrangements, namely, centric fusions and tandem fusions [41]. Such rearrangements were previously detected in various teleost species (reviewed in Ocalewicz [42]). However, many cases of ancestral chromosome fusions may not have the expected ITS, probably due to loss or drastic reduction of the telomeric DNA during the rearrangements that followed these events [43]. The absence of ITSs and the same 2n in studied species may indicate the absence of structural rearrangements involving terminal regions in Melanotaeniidae karyotype evolution.

In conclusion, our study provides the first cytogenetic analyses of representatives of all five lineages of the family Melanotaeniidae by combining conventional and molecular cytogenetic approaches. Despite the conservatism in chromosome number, cytogenetic differences in the topology of chromosome markers (constitutive heterochromatin regions, rDNA sites, GC-rich regions) were found among different genera. The observed differences correlate with the degree of species divergence and might be associated with chromosomal rearrangements, known to play a fundamental role in speciation [44,45]. Nevertheless, additional detailed cytogenetic studies on a wider taxonomic scale are still needed for detailed description of the karyotype evolution in the family Melanotaeniidae.

## Figures and Tables

**Table 1:** Number of individuals and metaphases analysed in this study.

Species	No. of Individuals	No. of Metaphases Examined				
		Giemsa	C Banding	CMA_3_	Telomeres	FISH
*Melanotaenia* sp.	3 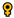 ; 3 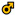	50	13	31	10	15
*Glossolepis incisus*	2 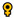 ; 2 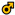	22	21	13	12	16
*Iriatherina werneri*	2 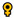 ; 5 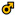	25	35	30	35	28
*Rhadinocentrus ornatus*	2 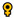 ; 2 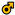	40	14	10	59	22
*Cairnsichthys rhombosomoides*	2 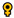 ; 2 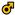	80	40	15	14	28

CMA3—chromomycin A3, FISH—fluorescence in situ hybridisation.

**Table 2:** Summary of cytogenetic results. NF—fundamental number; rDNA—ribosomal DNA.

Species	2n	Karyotype Composition	NF	28S rDNA Clusters	5S rDNA Clusters	Telomeres	AT-Rich/GC-Rich Regions	Heterochromatin Regions
*Melanotaenia* sp.	48	48st/a	48	2	4	no ITS	2 pericenromeric	32 centromeric
*Glossolepis incisus*	48	48st/a	48	2	4	no ITS	2 pericenromeric	14 centromeric
*Iriatherina werneri*	48	48st/a	48	2	4	no ITS	2 pericenromeric	36 centromeric 4 interstitial
*Rhadinocentrus ornatus*	48	12sm + 36st/a	60	2	4	no ITS	2 telomeric	28 centromeric 2telomeric
*Cairnsichthys rhombosomoides*	48	8sm + 40st/a	56	2	8	no ITS	2 pericenromeric	8 centromeric 4 interstitial 2 large clusters

## Supplementary Materials

The following are available online at https://www.mdpi.com/2073-4425/11/7/818/s1: Figure S1. Male and female karyotypes of all studies species.
