# Supplementary material for: Evidence of Interspecific Chromosomal Diversification in Rainbowfishes (Melanotaeniidae, Teleostei)
Source: Genes (Basel). 2020 Jul 18;11(7):818. doi: 10.3390/genes11070818 (PMC7397213; doi:10.3390/genes11070818)
Supplement: Supplementary file 1 [file genes-11-00818-s001.pdf]

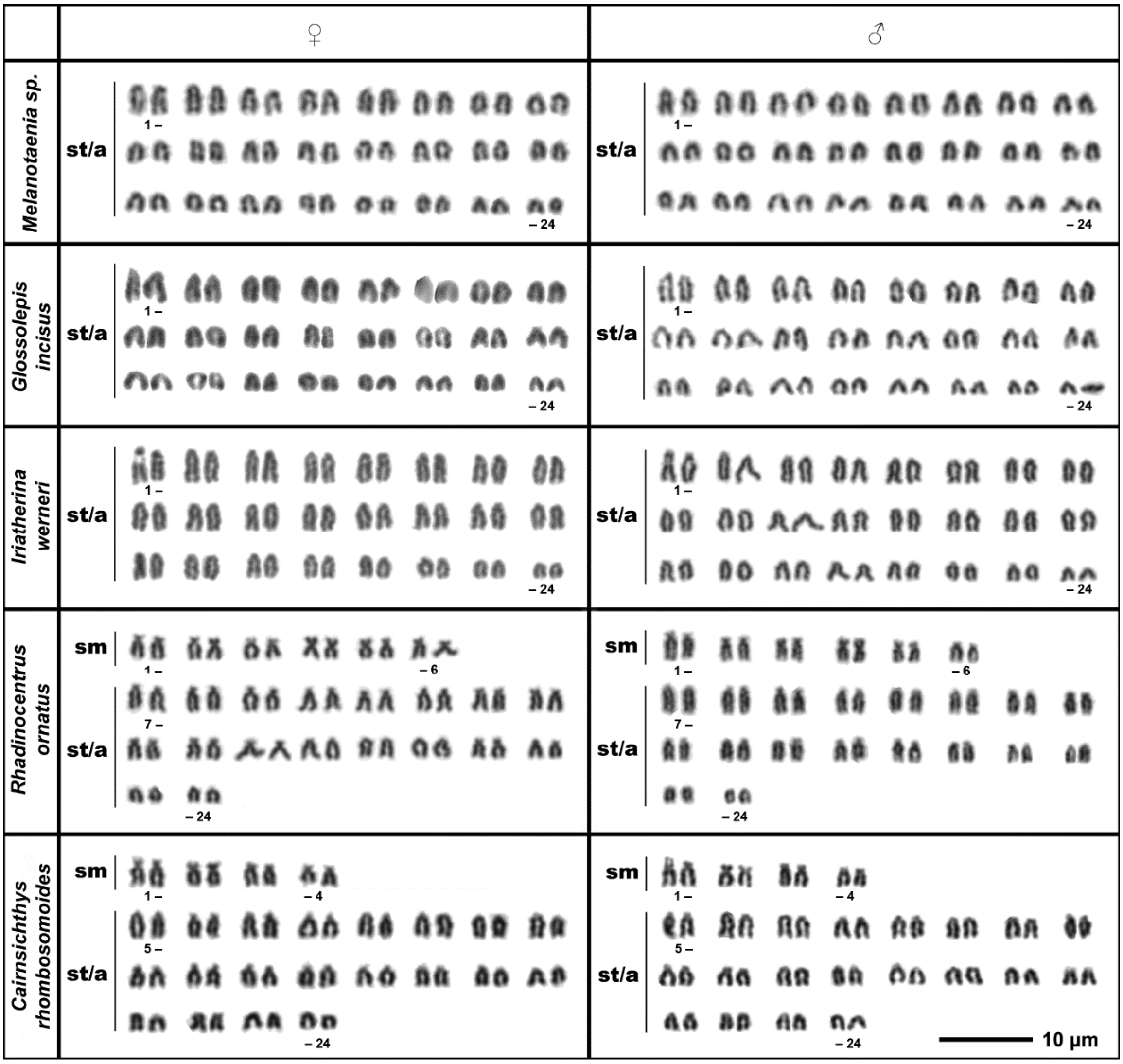

Supplementary Figure 1. Male and female karyotypes of all studies species. sm, submetacentric; st/a, subtelocentric-acrocentric chromosomes. Bar equals 10 µm.
